# Pediatric Lipid Screening Prevalence Using Nationwide Electronic Medical Records

**DOI:** 10.1001/jamanetworkopen.2024.21724

**Published:** 2024-07-23

**Authors:** Angela M. Thompson-Paul, Emily M. Kraus, Renee M. Porter, Samantha L. Pierce, Lyudmyla Kompaniyets, Ahlia Sekkarie, Alyson B. Goodman, Sandra L. Jackson

**Affiliations:** 1 Division for Heart Disease and Stroke Prevention, National Center for Chronic Disease Prevention and Health Promotion, Centers for Disease Control and Prevention, Atlanta, Georgia; 2 US Public Health Service Commissioned Corps, Rockville, Maryland; 3 Division of Nutrition, Physical Activity, and Obesity, National Center for Chronic Disease Prevention and Health Promotion, Centers for Disease Control and Prevention, Atlanta, Georgia; 4 Public Health Informatics Institute, Task Force for Global Health, Decatur, Georgia; 5 Kraushold Consulting, Denver, Colorado; 6 Epidemic Intelligence Service, Centers for Disease Control and Prevention, Atlanta, Georgia

## Abstract

**Importance:**

Universal screening to identify unfavorable lipid levels is recommended for US children aged 9 to 11 years and adolescents aged 17 to 21 years (hereafter, *young adults*); however, screening benefits in these individuals have been questioned. Current use of lipid screening and prevalence of elevated lipid measurements among US youths is not well understood.

**Objective:**

To investigate the prevalence of ambulatory pediatric lipid screening and elevated or abnormal lipid measurements among US screened youths by patient characteristic and test type.

**Design, Setting, and Participants:**

This cross-sectional study used data from the IQVIA Ambulatory Electronic Medical Record database and included youths aged 9 to 21 years with 1 or more valid measurement of height and weight during the observation period (2018-2021). Body mass index (BMI) was calculated and categorized using standard pediatric BMI percentiles (9-19 years) and adult BMI categories (≥20 years). The data were analyzed from October 6, 2022, to January 18, 2023.

**Main Outcomes and Measures:**

Lipid measurements were defined as abnormal if 1 or more of the following test results was identified: total cholesterol (≥200 mg/dL), low-density lipoprotein cholesterol (≥130 mg/dL), very low-density lipoprotein cholesterol (≥31 mg/dL), non–high-density lipoprotein cholesterol (≥145 mg/dL), and triglycerides (≥100 mg/dL for children aged 9 years or ≥130 mg/dL for patients aged 10-21 years). After adjustment for age group, sex, race and ethnicity, and BMI category, adjusted prevalence ratios (aPRs) and 95% CIs were calculated.

**Results:**

Among 3 226 002 youths (23.9% aged 9-11 years, 34.8% aged 12-16 years, and 41.3% aged 17-21 years; 1 723 292 females [53.4%]; 60.0% White patients, 9.5% Black patients, and 2.4% Asian patients), 11.3% had 1 or more documented lipid screening tests. The frequency of lipid screening increased by age group (9-11 years, 9.0%; 12-16 years, 11.1%; 17-21 years, 12.9%) and BMI category (range, 9.2% [healthy weight] to 21.9% [severe obesity]). Among those screened, 30.2% had abnormal lipid levels. Compared with youths with a healthy weight, prevalence of an abnormal result was higher among those with overweight (aPR, 1.58; 95% CI, 1.56-1.61), moderate obesity (aPR, 2.16; 95% CI, 2.14-2.19), and severe obesity (aPR, 2.53; 95% CI, 2.50-2.57).

**Conclusions and Relevance:**

In this cross-sectional study of prevalence of lipid screening among US youths aged 9 to 21 years, approximately 1 in 10 were screened. Among them, abnormal lipid levels were identified in 1 in 3 youths overall and 1 in 2 youths with severe obesity. Health care professionals should consider implementing lipid screening among children aged 9 to 11 years, young adults aged 17 to 21 years, and all youths at high cardiovascular risk.

## Key Points

**Question:**

What is the prevalence of lipid screening and elevated or abnormal lipid measurements among US youths?

**Findings:**

In this cross-sectional study of data on 3 226 002 US youths aged 9 to 21 years from the IQVIA Ambulatory Electronic Medical Record database, 11% of patients had documented lipid screening and screening frequency was found to increase with age and body mass index. Among those screened, 30% had 1 or more abnormal test results, with the highest prevalence among those with moderate (42%) or severe (48%) obesity.

**Meaning:**

The findings of this study indicate that lipid screening frequency among youths may be low and that 1 in 3 individuals screened may have abnormal lipid levels.

## Teaser

This cross-sectional study uses data from the IQVIA Ambulatory Electronic Medical Record database to examine the prevalence of lipid screening and elevated or abnormal lipid measurements among US youths aged 9 to 21 years.

## Introduction

Atherosclerotic cardiovascular disease (CVD) begins in childhood and progresses through adolescence, partly driven by excess weight and unfavorable blood lipid trajectories.^1^ Cumulative exposure to unfavorable blood lipid levels increases CVD risk^1,2,3,4,5,6^; therefore, identifying dyslipidemia and intervening early with nonpharmacologic or pharmacologic treatment may be key to shifting lipid trajectories and reducing lifetime CVD risk.

Universal pediatric lipid screening may be an important tool in the early identification of children with elevated lipid levels who could benefit from earlier intervention. In children, lipid screening prevalence has been shown to differ by risk factor status,^7,8^ and mean cholesterol levels have been shown to differ by race and ethnicity.^8^ Studies have shown that lipid screening based on family history or risk factors alone failed to identify 30% to 60% of children with severe dyslipidemia.^9,10,11^ Family history may not be accurately reported and is a poor predictor of cholesterol levels in adolescents.^12^ In the 2011 Guidelines for Cardiovascular Health and Risk Reduction in Children and Adolescents,^13^ the National Heart, Lung, and Blood Institute (NHLBI) recommended universal lipid screening once in each period for children aged 9 to 11 years and young adults aged 17 to 21 years. The American Academy of Pediatrics (AAP) endorsed this recommendation.^14^ Targeted lipid screening is also recommended by the AAP for children and young adults with overweight and obesity as part of a risk factor evaluation.^15^ Children and young adults with obesity have increased prevalence of cardiometabolic risk factors, including dyslipidemia, than youths of healthy weight.^8,16^ Prevalence of abnormal lipid levels has also been shown to differ across race and ethnicity among youths.^8^

In 2016 and 2023, the US Preventive Services Task Force (USPSTF) conducted evidence reviews to examine lipid screening in children and adolescents.^17,18^ They were unable to identify any studies that demonstrated that screening for dyslipidemia in asymptomatic children or adolescents delayed or reduced the incidence of CVD or mortality, nor were they able to identify any studies that demonstrated harms of screening. They concluded that there is still insufficient evidence to assess the balance of benefits or harms of lipid screening among children.^17,18^

Despite these screening recommendations, studies have shown low pediatric lipid screening rates, including among youths with higher-risk conditions such as obesity.^7,10,19^ However, prior studies examining screening prevalence are limited in number and recency of data. We investigated the prevalence of lipid screening and dyslipidemia in a large sample of US outpatients aged 9 to 21 years from 2018 to 2021.

## Methods

This cross-sectional study explored the frequency of lipid screening tests conducted from 2018 to 2021 among US youths aged 9 to 21 years. Among those screened, we examined the prevalence of elevated or abnormal lipid levels. We followed the Strengthening the Reporting of Observational Studies in Epidemiology (STROBE) reporting guideline. This study was exempted from institutional review board approval by the Centers for Disease Control and Prevention because it was a public health surveillance study. According to Privacy Analytics, which approved the Reidentification Risk Determination, informed consent was not required since the patients in the dataset were deidentified through IQVIA's proprietary encryption process.

### Data Source

We used IQVIA’s Ambulatory Electronic Medical Record (AEMR) (version 5, May 2022 release) database, which contains US medical records from more than 100 000 physicians and more than 800 ambulatory practices and physician networks.^20^ We accessed and extracted data—including clinical diagnoses, medication orders, and laboratory test results—using the E360 Software-as-a-Service Platform (OMOP version 5, February 2023 data release). These data include patient year of birth, sex, race and ethnicity, and clinical variables such as encounter date, height and weight, diagnostic codes, laboratory results, procedures, and medication orders. Race and ethnicity were reported by patients or clinicians and available as a single composite variable in the dataset.

### Study Population and Characteristics

The study population included outpatients aged 9 to 21 years with 1 or more valid body mass index (BMI, calculated as weight in kilograms divided by height in meters squared) measurement during 2018 to 2021. The data were analyzed from October 6, 2022, to January 18, 2023. We calculated each patient’s age at the midpoint of their birth year (ie, July 2) and categorized it into the following age groups: 9 to 11, 12 to 16, and 17 to 21 years to align with guideline-recommended screening ages. Prior to selection of the visit used in the analysis, we cleaned all height and weight values using growthcleanr.^21,22^ We used these cleaned height and weight values to calculate BMI and BMI percentile; extreme BMI values were excluded (eMethods in Supplement 1). For patients aged 9 to 19 years, BMIs were categorized based on sex- and age-specific percentiles: underweight (<5th percentile), healthy weight (5th to <85th percentile), overweight (≥85th to <95th percentile), moderate obesity (≥95th percentile to <120% of the 95th percentile), and severe obesity (≥120% of the 95th percentile).^23^ For patients aged 20 to 21 years, BMIs were categorized based on adult cut points: underweight (BMI <18.5), healthy weight (BMI ≥18.5 and <25), overweight (BMI ≥25 and <30), moderate obesity (BMI ≥30 and <40), and severe obesity (BMI ≥40).^24^

Patients were assigned to an age, age group, and BMI category as follows. Patients without lipid screening results were assigned to an age and BMI category based on the date of the earliest BMI value during the observation period. Patients with 1 or more lipid screening results were assigned to an age category based on the date of the first lipid screening result during the observation period and to a BMI category based on the BMI collection date closest to the first lipid screening result. Each unique person included in the analysis may have multiple test results (ie, ≥1 test in a lipid panel or ≥1 lipid panel). The analysis examined for any evidence of elevated values; therefore, if an individual had 1 low-density lipoprotein (LDL) test result in the healthy range and 1 in the elevated range, they would be categorized as having an elevated LDL level.

### Screening Tests and Results

We used a 4-year observation period (January 1, 2018, to December 31, 2021) for this study. The NHLBI recommends screening for dyslipidemia with a panel of lipid tests for low-density lipoprotein cholesterol (LDL-C), very low-density lipoprotein cholesterol, triglycerides, non–high-density lipoprotein cholesterol (non–HDL-C), and total cholesterol (TC), hereafter referred to as *screening tests* for dyslipidemia and their results as *screening results*. Current recommendations for children and young adults allow screening to be performed with a fasting or a nonfasting plasma lipid profile to document baseline LDL-C level.^14^

Biologically implausible lipid values were excluded, and the results were categorized as healthy or elevated (with elevated measures subdivided into borderline or abnormal) based on the 2018 multisociety Guideline on the Management of Blood Cholesterol thresholds (Table 1).^14^ We then identified patients with 1 or more elevated or 1 or more abnormal lipid screening results. Lipid measurements were defined as borderline if any of the following test results was identified: TC, 170 to 199 mg/dL; LDL-C, 110 to 129 mg/dL; non–HDL-C, 120 to 144 mg/dL; or triglycerides, 75 to 99 mg/dL for children aged 0 to 9 years or 90 to 129 mg/dL for patients aged 10 to 21 years. They were defined as abnormal if 1 or more of the following was identified: TC, 200 mg/dL or higher; LDL-C, 130 mg/dL or higher; very LDL-C, 31 mg/dL or higher; non–HDL-C, 145 mg/dL or higher; and triglycerides, 100 mg/dL or higher for children 0 to 9 years or 130 mg/dL or higher for patients 10 to 21 years. (To convert all cholesterol measurements to mmol/L, multiply by 0.0259; triglycerides to mmol/L, multiply by 0.0113.)

**Table 1.** Thresholds for Categorization of Lipid Screening Tests^a^

Screening test	Test results, mg/dL			
	Healthy	Elevated		Implausible
		Borderline	Abnormal	
Total cholesterol	1-169	170-199	200-2000	>2000
Triglycerides				
Patients aged 0-9 y	1-74	75-99	100-100 000	>100 000
Patients aged 10-21 y	1-89	90-129	130-100 000	>100 000
Low-density lipoprotein cholesterol	1-109	110-129	130-2000	>2000
Non–high-density lipoprotein cholesterol	1-119	120-144	145-2000	>2000
Very low-density lipoprotein cholesterol	1-30	NA	31-2000	>2000

Abbreviation: NA, not applicable

SI conversion factors: To convert cholesterol to mmol/L, multiply by 0.0259; triglycerides to mmol/L, multiply by 0.0113.

^a^ Information taken from Grundy et al.^14^

We calculated the proportion of patients with screening results (screening prevalence) and, among those with screening results, we calculated the proportion of patients with 1 or more elevated or 1 or more abnormal results, including 95% CIs. Among all lipid screening results, we examined the proportion of results that were in the healthy, elevated, or abnormal ranges by each screening test type.

### Statistical Analysis

Descriptive statistics for patients were summarized by age group, sex, race and ethnicity, and BMI category; 95% CIs were calculated for proportions. Multivariable logit models were used to assess the association between patient characteristics and lipid screening (model 1), elevated screening test results (model 2), and abnormal lipid screening results (model 3) as the outcome variables. Models 2 and 3 were restricted to patients who were screened. All models included the following covariates: age group (9-11 years [reference], 12-16 years, and 17-21 years), sex (male [reference], female), race and ethnicity groups (Asian, Black, Hispanic, other, unknown, and White [reference]), and BMI category (underweight, healthy weight [reference], overweight, moderate obesity, and severe obesity). The other race and ethnicity category may include American Indian or Alaska Native, Native Hawaiian or Other Pacific Islander, self-reported other, and other groups. It is unknown if patients self-reported the other race and ethnicity category or if IQVIA combined them.

We obtained the estimated probability of an outcome at each level of every covariate (P1) and at the reference level of each covariate (P0). Adjusted prevalence ratio (aPR) was estimated as the ratio of P1:P0.^25,26^ All data cleaning and analyses were conducted using Stata, version 15.1 (StataCorp LLC); R, version 4.2.3 (R Project for Statistical Computing); and SAS, version 9.3 (SAS Institute Inc).

## Results

The study cohort included 3 226 002 patients (1 723 292 [53.4%] were females and 1 502 710 [46.6%] were males; 23.9% were aged 9-11 years, 34.8% were 12-16 years, and 41.3% were 17-21 years). Among the patients, 2.4% were Asian; 9.5%, Black; 0.7%, Hispanic; 60.0%, White; 4.2%, other; and 23.1%, unknown. Of the total patients, 3.5% had underweight, 56.5% had a healthy weight, 18.2% had overweight, 14.1% had moderate obesity, and 7.7% had severe obesity (Table 2). Lipid screening results were identified for 11.3% (n = 366 109) of the study population. Prevalence of lipid screening differed by age group, race and ethnicity, and BMI category (Table 2). Screening frequency increased with age (9-11 years, 9.0%; 12-16 years, 11.1%; 17-21 years, 12.9%) and BMI category (range, 9.2% [healthy weight] to 21.9% [severe obesity]). Compared with patients aged 9 to 11 years, prevalence of lipid screening was higher among older age groups (12-16 years: aPR, 1.24; 95% CI, 1.22-1.25; 17-21 years: aPR, 1.46; 95% CI, 1.45-1.47). Compared with White patients, individuals more likely to undergo a lipid screening were Asian patients (aPR, 1.83; 95% CI, 1.80-1.85) and Black patients (aPR, 1.26; 95% CI, 1.25-1.27). Compared with patients with a healthy weight, patients in all other BMI categories were more likely to be screened (severe obesity: aPR, 2.35; 95% CI, 2.33-2.37; moderate obesity: aPR, 1.58; 95% CI, 1.57-1.59; overweight: aPR, 1.18; 95% CI, 1.17-1.19; and underweight: aPR, 1.05; 95% CI, 1.03-1.07).

**Table 2.** Crude and Estimated Prevalence of Lipid Screening by Demographic Attribute and BMI Category, IQVIA Ambulatory Electronic Medical Record, 2018-2021

Demographic attribute	No. (%)		Adjusted^a^	
	Study population	Patients with ≥1 lipid screening result^b^	Estimated lipid screening prevalence, % (95% CI)	Prevalence ratio (95% CI)
Overall	3 226 002 (100)	366 109 (11.3)	11.3 (11.3-11.4)	NA
Age group, y				
9-11	771 618 (23.9)	69 706 (9.0)	8.9 (8.9-9.0)	1 [Reference]
12-16	1 122 662 (34.8)	124 420 (11.1)	11.0 (11.0-11.1)	1.24 (1.22-1.25)^c^
17-21	1 331 722 (41.3)	171 983 (12.9)	13.0 (13.0-13.1)	1.46 (1.45-1.47)^c^
Sex				
Female	1 723 292 (53.4)	195 443 (11.3)	11.3 (11.2-11.3)	0.98 (0.98-0.99)^c^
Male	1 502 710 (46.6)	170 666 (11.4)	11.5 (11.4-11.5)	1 [Reference]
Race and ethnicity^d^				
Asian	78 178 (2.4)	14 231 (18.2)	20.0 (19.7-20.3)	1.83 (1.80-1.85)^c^
Black	306 553 (9.5)	44 509 (14.5)	13.8 (13.7-13.9)	1.26 (1.25-1.27)^c^
Hispanic	22 213 (0.7)	2517 (11.3)	10.8 (10.4-11.2)	1.00 (0.96-1.03)
White	1 936 175 (60.0)	209 793 (10.8)	10.9 (10.9-11.0)	1 [Reference]
Other^e^	136 821 (4.2)	20 478 (15.0)	14.6 (14.4-14.8)	0.99 (0.95-1.02)
Unknown	746 062 (23.1)	74 581 (10.0)	9.9 (9.8-10.0)	1.34 (1.32-1.35)^c^
BMI category^f^				
Underweight	113 368 (3.5)	11 281 (10.0)	9.8 (9.6-9.9)	1.05 (1.03-1.07)^c^
Healthy weight	1 822 742 (56.5)	168 459 (9.2)	9.3 (9.2-9.3)	1 [Reference]
Overweight	585 892 (18.2)	64 815 (11.1)	11.0 (10.9-11.1)	1.18 (1.17-1.19)^c^
Moderate obesity	454 284 (14.1)	66 894 (14.7)	14.7 (14.6-14.8)	1.58 (1.57-1.59)^c^
Severe obesity	249 716 (7.7)	54 660 (21.9)	21.9 (21.7-22.0)	2.35 (2.33-2.37)^c^

Abbreviations: BMI, body mass index (calculated as weight in kilograms divided by height in meters squared); NA, not applicable.

^a^ The adjusted columns represent the results from a single logit model, with abnormal lipid result (yes or no) as the outcome variable and the following covariates: age group, sex, race and ethnicity, and BMI category. The estimated probability of screening was obtained at each level of every covariate (P1) and at the reference level of each covariate (P0). The prevalence ratio was estimated as the ratio of P1:P0.

^b^ Lipid screening results include results from low-density lipoprotein cholesterol, very low-density lipoprotein cholesterol, non–high-density lipoprotein cholesterol, triglycerides, or total cholesterol, as identified in IQVIA through a keyword search.

^c^ Statistically significant because the 95% CI does not cross 1.0.

^d^ IQVIA combines race and ethnicity in 1 field, which may cause loss of true racial ethnic identity data at the patient level.

^e^ Other may include American Indian or Alaska Native, Native Hawaiian or Other Pacific Islander, self-reported other, and other groups. It is unknown if patients self-reported the other race and ethnicity group or IQVIA combined them.

^f^ Assigned based on earliest BMI for patients without lipid screening results or based on BMI closest to first lipid screening for patients with lipid screening results. The BMI category for patients aged 9 to 19 years is defined as underweight (<5th percentile), healthy weight (5th to <85th percentile), overweight (≥85th to <95th percentile), moderate obesity (≥95th percentile and <120% of 95th percentile), and severe obesity (≥120% of 95th percentile). The BMI category for patients aged 20 to 21 years is defined as underweight (BMI <18.5), healthy weight (BMI ≥18.5 and <25), overweight (BMI ≥25 and <30), moderate obesity (BMI ≥30 and <40), and severe obesity (BMI ≥40).

Among patients with lipid screening results, 217 004 (59.3%) had 1 or more elevated results and 110 567 (30.2%) had 1 or more abnormal results (Table 3). Prevalence of elevated results differed by age group, sex, race and ethnicity, and BMI. The prevalence of elevated lipid screening results was highest among patients aged 9 to 11 years compared with that for older age groups (12-16 years: aPR, 0.91; 95% CI, 0.91-0.92; 17-21 years: aPR, 0.98; 95% CI, 0.97-0.99) and among females (aPR, 1.04; 95% CI, 1.03-1.04) compared with males. Compared with White youths, prevalence of elevated lipid levels was higher among Asian youths (aPR, 1.07; 95% CI, 1.06-1.09) and lower among Black youths (aPR, 0.77; 95% CI, 0.77-0.78). Prevalence of elevated lipid levels was lower among youths with underweight (aPR, 0.87; 95% CI, 0.85-0.89) compared with those with healthy weight. Prevalence was higher among higher BMI categories compared with healthy weight (overweight: aPR, 1.25; 95% CI, 1.24-1.26; moderate obesity: aPR, 1.46; 95% CI, 1.45-1.47; and severe obesity: aPR, 1.59; 95% CI, 1.58-1.60). The magnitude of association for prevalence of abnormal lipid screening results was similar to the association observed in the elevated results for age group, sex, and race and ethnicity but increased for BMI category. The prevalence of abnormal lipid results was highest among those with overweight and obesity (overweight: aPR, 1.58; 95% CI, 1.56-1.61; moderate obesity: aPR, 2.16; 95% CI, 2.14-2.19; severe obesity: aPR, 2.53; 95% CI, 2.50-2.57) compared with individuals with healthy weight.

**Table 3.** Prevalence of Elevated Lipid Levels Among Patients With Documented Screening Results by Demographic Characteristics, IQVIA Ambulatory Electronic Medical Record, 2018-2021

Demographic characteristic	Elevated lipid screening results			Abnormal lipid screening results		
	Patients with ≥1 elevated lipid screening result, No. (%)^a^	Estimated prevalence (95% CI)^b^	Prevalence ratio (95% CI)^b^	Patients with ≥1 abnormal lipid screening result, No. (%)^a^	Estimated prevalence (95% CI)^b^	Prevalence ratio (95% CI)^b^
Total	217 004 (59.3)	59.3 (59.1-59.4)	NA	110 567 (30.2)	30.2 (30.1-30.3)	NA
Age group, y						
9-11	43 093 (61.8)	61.6 (61.3-62.0)	1 [Reference]	22 518 (32.3)	31.9 (31.6-32.2)	1 [Reference]
12-16	71 351 (57.4)	56.4 (56.1-56.6)	0.91 (0.91-0.92)^c^	36 167 (29.1)	28.0 (27.8-28.3)	0.88 (0.87-0.89)^c^
17-21	102 560 (59.6)	60.4 (60.2-60.6)	0.98 (0.97-0.99)^c^	51 882 (30.2)	31.1 (30.9-31.3)	0.98 (0.96-0.99)^c^
Sex						
Female	117 564 (60.2)	60.3 (60.1-60.5)	1.04 (1.03-1.04)^c^	57 929 (29.6)	29.9 (29.7-30.1)	0.98 (0.97-0.99)^c^
Male	99 440 (58.3)	58.1 (57.9-58.3)	1 [Reference]	52 638 (30.8)	30.6 (30.4-30.8)	1 [Reference]
Race and ethnicity^d^						
Asian	8792 (61.8)	65.7 (65.0-66.5)	1.07 (1.06-1.09)^c^	4298 (30.2)	34.9 (34.2-35.7)	1.09 (1.06-1.11)^c^
Black	22 262 (50.0)	47.4 (46.9-47.8)	0.77 (0.77-0.78)^c^	9348 (21.0)	19.1 (18.7-19.4)	0.59 (0.58-0.60)^c^
Hispanic	1530 (60.8)	59.0 (57.2-60.9)	0.96 (0.93-1.00)	804 (31.9)	30.0 (28.3-31.7)	0.93 (0.88-0.99)^c^
White	127 646 (60.8)	61.2 (61.0-61.4)	1 [Reference]	66 470 (31.7)	32.2 (32.0-32.3)	1 [Reference]
Other^e^	13 055 (63.8)	61.8 (61.2-62.5)	1.01 (1.00-1.02)	7233 (35.3)	33.2 (32.6-33.8)	1.03 (1.01-1.05)^c^
Unknown	43 719 (58.6)	58.7 (58.3-59.0)	0.96 (0.95-0.96)^c^	22 414 (30.1)	30.1 (29.7-30.4)	0.94 (0.92-0.95)^c^
BMI category^f^						
Underweight	4890 (43.3)	42.5 (41.6-43.4)	0.87 (0.85-0.89)^c^	1722 (15.3)	14.7 (14.1-15.4)	0.75 (0.71-0.78)^c^
Healthy weight	83 013 (49.3)	48.8 (48.6-49.1)	1 [Reference]	33 649 (20.0)	19.7 (19.5-19.9)	1 [Reference]
Overweight	39 723 (61.3)	61.1 (60.7-61.4)	1.25 (1.24-1.26)^c^	20 289 (31.3)	31.2 (30.8-31.5)	1.58 (1.56-1.61)^c^
Moderate obesity	47 545 (71.1)	71.3 (70.9-71.6)	1.46 (1.45-1.47)^c^	28 401 (42.5)	42.7 (42.3-43.0)	2.16 (2.14-2.19)^c^
Severe obesity	41 833 (76.5)	77.6 (77.3-78.0)	1.59 (1.58-1.60)^c^	26 506 (48.5)	50.0 (49.6-50.4)	2.53 (2.50-2.57)^c^

Abbreviations: BMI, body mass index (calculated as weight in kilograms divided by height in meters squared); NA, not available.

^a^ Lipid screening test results include results from low-density lipoprotein cholesterol, very low-density lipoprotein cholesterol, non–high-density lipoprotein cholesterol, triglycerides, or total cholesterol as identified in IQVIA through a keyword search.

^b^ The adjusted columns represent the results from a single logit model, with elevated or abnormal lipid result (yes/no) as the applicable outcome variable and the following covariates: age group, sex, race and ethnicity, and BMI category. The estimated probability of screening was obtained at each level of every covariate (P1) and at the reference level of each covariate (P0). Adjusted prevalence ratio was estimated as the ratio of P1:P0.

^c^ Statistically significant because the 95% CI does not cross 1.0.

^d^ IQVIA combines race and ethnicity in 1 field, which may cause loss of true racial and ethnic identity data at the patient level.

^e^ Other may include American Indian or Alaska Native, Native Hawaiian or Other Pacific Islander, self-reported other, and other groups. It is unknown if patients self-reported the other race and ethnicity group or IQVIA combined them.

^f^ Assigned based on BMI closest to first lipid screening result. See the footnote to Table 2 for definitions of the BMI categories.

**Figure 1.**
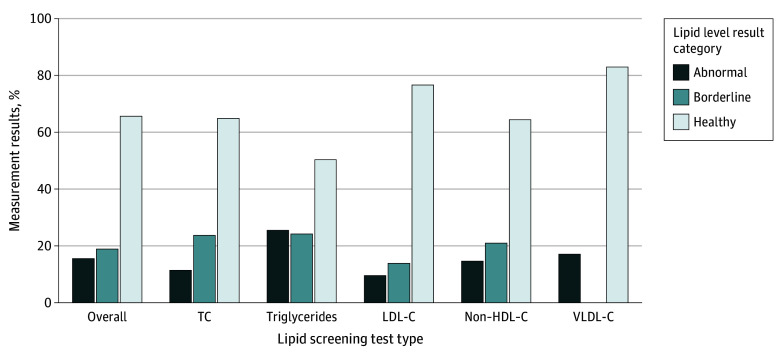
Lipid Screening by Test Type and Result Category, IQVIA Ambulatory Electronic Medical Records (2018-2021) Lipid measurements were considered elevated if 1 or more of the following results were obtained: total cholesterol (TC), 170 mg/dL or higher; low-density lipoprotein cholesterol (LDL-C), 110 mg/dL or higher; very low-density lipoprotein cholesterol (VLDL-C), 31 mg/dL or higher, non–high-density lipoprotein cholesterol (HDL-C), 120 mg/dL or higher; or triglycerides, 75 mg/dL or higher (9 years) or 90 mg/dL or higher (10-21 years). Among the elevated values, levels were defined as abnormal if 1 or more of the following were obtained: TC, 200 mg/dL or higher; LDL-C, 130 mg/dL or higher; VLDL-C, 31 mg/dL or higher; non–HDL-C, 145 mg/dL or higher; or triglycerides, 100 mg/dL or higher (9 years) or 130 mg/dL or higher (10-21 years). (To convert all cholesterol measurements to mmol/L, multiply by 0.0259; triglycerides to mmol/L, multiply by 0.0113.)

**Figure 2.**
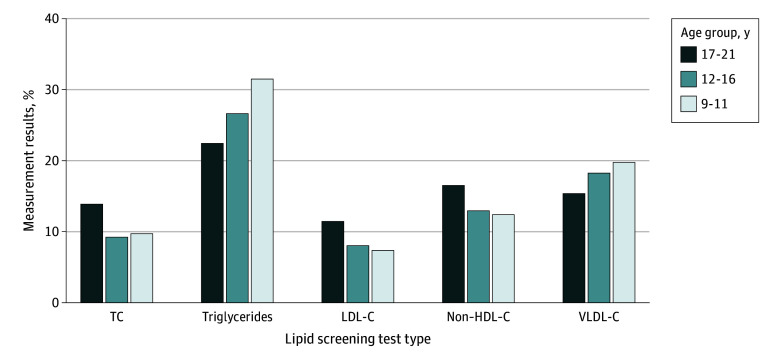
Abnormal Lipid Screening Results by Test Type and Age Group, IQVIA Ambulatory Electronic Medical Records (2018-2021) See the caption to Figure 1 for expansions of the abbreviations and for definitions of abnormal lipid measurements and the lipid level categories.

Overall, 65.6% of measurement results were in the healthy range; 18.9%, borderline; and 15.5%, abnormal (Figure 1). Abnormal results were observed the most frequently for triglycerides (25.5% of the screened population); however, 11.4% of TC and 9.5% of LDL-C test results were abnormal. The proportions of abnormal TC, LDL-C, and non–HDL-C test results were moderately higher among patients aged 17 to 21 years compared with younger age groups, while prevalence of abnormal triglycerides was highest in the youngest age group (Figure 2). The overall proportion of abnormal test results differed by age group (eTable 1 in Supplement 1) and across race and ethnicity (eTable 2 in Supplement 1). A greater proportion of Black patients were screened, but a lower proportion had abnormal screening test results compared with White patients. A greater proportion of Asian patients were also screened and found to be more likely to have elevated or abnormal results than White patients.

## Discussion

This cross-sectional study indicated a low prevalence of lipid screening among US children aged 9 to 11 years, young adults aged 17 to 21 years, and youths with established risk factors, such as obesity. Approximately 1 in 10 youths (9-11 years [9.0%]; 12-16 years [11.1%]; 17-21 years [12.9%]) had documented lipid screening during 2018-2021. Among the individuals screened, nearly 2 in 3 had an elevated result, and approximately 1 in 3 had an abnormal result. Screening prevalence was greater among higher BMI categories; nonetheless, fewer than 1 in 5 patients with obesity were screened. Of note, individuals with overweight or obesity were 1.5 to 2.5 times more likely to have an abnormal test result compared with those with healthy weight. Abnormal lipid test results were most frequent for triglycerides (25.5% of the screened patients); however, 11.4% of TC and 9.5% of LDL-C test results were abnormal.

Our findings are concordant with previous studies,^7,10,19^ which have found low lipid screening prevalence in children, including in high-risk patients. In 1 large health care system, only 4.0% of eligible patients aged 9 to 11 years were screened.^19^ In a study using MarketScan commercial and Medicaid insurance claims data (>500 000 patients), Berger and colleagues^7^ reported that 20% of youths at standard risk and 47% of those at high risk were screened. The MarketScan database includes both (1) commercial claims information from health care encounters from patients in participating health insurance plans and (2) data from a limited number of states with Medicaid insurance. Berger and colleagues^7^ excluded patients with discontinuous insurance coverage during the study years. As such, their study sample may include a healthier population with better health care insurance coverage than the IQVIA AEMR (which includes all payers). Claims data among persons with continuous insurance coverage may include more comprehensive screening data compared with the AEMR from a single health care professional (which could miss a patient’s records from other clinicians).

A study using data from 3 large US health systems,^27^ prior to the release of the 2011 guidelines, found that lipid screening occurred in 5.9% of children with healthy weight, 10.8% with overweight, and 26.9% with obesity; and by age, screening occurred in 8.9% of children aged 9 to 11 years and 24.3% of individuals aged 17 to 19 years. Our study found a similar prevalence of screening indicating that despite recommendations, screening practices might not have improved in more than a decade; however, it is important to note that neither study included nationally representative samples.

A high proportion of US youths have abnormal lipid levels, including children and young adults with healthy weight and excess weight.^28^ Using fasting measurements from the National Health and Nutrition Examination Survey, Perak and colleagues^29^ reported that 19.2% of youths aged 9 to 19 years had abnormal TC, non–HDL-C, or LDL-C levels. In the present study, screening identified abnormal test results in 1 in 3 youths screened: approximately 1 in 10 youths had abnormal TC or LDL-C, 1 in 4 had abnormal triglycerides, and 1 in 7 had abnormal non–HDL-C levels. Our findings showed some differences by age group and by race and ethnicity. Similar to our findings, Nguyen and colleagues^8^ reported that non-Hispanic Asian children and adolescents had the highest prevalence of abnormal levels of TC and non–HDL-C compared with youths of other races and ethnicities.

Exposure to lower cholesterol levels in childhood is associated with better CVD outcomes in adulthood.^1^ Being screened at the recommended intervals during childhood and adolescence could contribute to primary CVD prevention through increased awareness and behavior change or pharmacologic treatment. Our study adds observational evidence that a large proportion of youths who receive screening have elevated lipid levels and might benefit from early intervention. This is a missed opportunity for referring children and families to guideline-recommended treatment, which includes lifestyle modification and the use of lipid-lowering medication.^8^

Research on barriers to screening could help inform efforts to improve pediatric cholesterol screening rates.^10,30^ Our study revealed that screening levels are low among youths for whom screening is recommended (11.4%). Interestingly, a similar prevalence of screening occurred among adolescents aged 12 to 16 years for whom screening is not recommended (11.1%). Although universal lipid screening recommendations have been endorsed by the AAP since 2011, our findings may indicate that (1) health care professionals may not have a clear understanding of the childhood screening guidelines or (2) there may be barriers to changing screening and prevention practices.^31,32^ The USPSTF evidence reviews from 2016 and 2023 summarized randomized clinical trial data and found no direct evidence of either effectiveness or harms of universal lipid screening in youths.^17,18^ In contrast to AAP recommendations endorsing universal lipid screening, the USPSTF conclusion likely added confusion for clinicians regarding the necessity of cholesterol screening, which may negatively impact screening rates.

Improving health care professionals’ knowledge and beliefs about cholesterol screening guidelines for youths, as well as ensuring that clinicians feel supported to initiate screening and empowered to act on the screening results, may improve screening rates.^30^ Electronic health record modifications (eg, clinical decision support tools) may also improve screening rates among recommended groups. One study^10^ revealed that (1) the display of age-appropriate lipid reference ranges, (2) an alert reminding clinicians when screening is due, and (3) the addition of lipid screening to a list of recommended activities on the well-child visit template led to an increase in lipid screening orders from 8.9% to 50.0% over 12 months and screening prevalence of 46.8% over a 6-year period.

### Limitations

This study has several limitations. First, although IQVIA AEMR comprises a geographically diverse US patient population, the data are not nationally representative, and they characterize health care–seeking persons only. Second, this analysis relied on laboratory test results, which were included only if the facility performing the test contributed data to IQVIA, the test result was available in a structured or standardized format, and the test result could be identified using the IQVIA search tools. Otherwise, results may appear as incomplete or false-negatives for lipid screening and could lead to an underestimation of screening in this population. It is also possible that testing may have occurred outside the time frame of this study. Although our large sample size lends strength to our findings, they may underestimate the prevalence of screenings or abnormal results. Incomplete information was available regarding the fasting state of patients; however, the 2018 cholesterol guideline^14^ states that nonfasting lipid testing is effective for screening purposes. Our method of assigning age and BMI was systematic but may have resulted in misclassification of some patients into an older or younger age group, which could have subsequently also caused misclassification of BMI. Finally, race and ethnicity are presented as a single variable in the AEMR, and for 23% of patients, this variable was unknown. This high proportion led to an unclear composition of race and ethnicity in our study population; therefore, our race and ethnicity findings are challenging to interpret and may not be generalizable.

## Conclusions

To our knowledge, this cross-sectional study reflects the largest study population used to estimate pediatric dyslipidemia screening prevalence, using data from health care–seeking youths at more than 100 000 US clinical settings. The findings indicate that adherence to lipid screening recommendations among youths is low and that 1 in 3 of those screened have abnormal lipid levels, even higher among those with excess weight. Low lipid screening levels are a missed opportunity for both early treatment and longer-term prevention of adverse cardiovascular outcomes.

## Notes

Supplement 1. **eMethods.** Description of IQVIA AEMR Data Cleaning: Height and Weight **eTable 1.** Lipid Screening Results by Age Group and Lipid Screening Test Type, IQVIA 2018-2021 **eTable 2.** Comparison of Elevated Lipid Screening Results by Race and Ethnicity

Supplement 2.
Data Sharing Statement
