# Supplementary material for: Pediatric Lipid Screening Prevalence Using Nationwide Electronic Medical Records
Source: JAMA Netw Open. 2024 Jul 23;7(7):e2421724. doi: 10.1001/jamanetworkopen.2024.21724 (PMC11267408; doi:10.1001/jamanetworkopen.2024.21724)
Supplement: Supplement 1. — eMethods. Description of IQVIA AEMR Data Cleaning: Height and Weight eTable 1. Lipid Screening Results by Age Group and Lipid Screening Test Type, IQVIA 2018-2021 eTable 2. Comparison of Elevated Lipid Screening Results by Race and Ethnicity [file jamanetwopen-e2421724-s001.pdf]

Supplementary Online Content

Thompson-Paul AM, Kraus EM, Porter RM, et al. Pediatric lipid screening prevalence using nationwide electronic medical records. *JAMA Netw Open*. 2024;7(7):e2421724. doi:10.1001/jamanetworkopen.2024.21724

eMethods. Description of IQVIA AEMR Data Cleaning: Height and Weight

eTable 1. Lipid Screening Results by Age Group and Lipid Screening Test Type, IQVIA 2018-2021

eTable 2. Comparison of Elevated Lipid Screening Results by Race and Ethnicity

This supplementary material has been provided by the authors to give readers additional information about their work.

© 2024 Thompson-Paul AM et al. *JAMA Network Open.*

eMethods. Description of IQVIA AEMR Data Cleaning: Height and Weight

Erroneous height and weight measurements were identified using *growthcleanr,* a CRAN package for detecting errors in longitudinal growth data, based on the Daymont algorithm.[18,19] After removing erroneous measurements, we used the remaining height and weight values to calculate body mass index (BMI) for all patients and BMI percentile and extended BMI z-scores for pediatric patients.[28] We further excluded values of height, weight, BMI, and BMI z-scores that were higher or lower than expected. For adults, excluded values were defined as: height < 44 inches (112 cm) or > 90 inches (229 cm); weight < 25 kg (55 lbs) or > 454 kg (1000 lbs); or BMI < 12 kg/m^2 or > 110 kg/m^2). For children, excluded values were defined as: modified weight-for-age z-score (MWAZ) ≤ -5 or ≥ 10, modified height-for-age z-score (MHAZ) ≤ -5 or ≥ 5, modified BMI z-score (MBMIZ) ≤ -4 or ≥ 10, or BMI ≥ 150th percentile for age and sex.[28]

© 2024 Thompson-Paul AM et al. *JAMA Network Open.*

eTable 1. Lipid Screening Results by Age Group and Lipid Screening Test Type, IQVIA 2018-2021

			All		Lipid Screening Test Type									
					Total Cholesterol		Triglycerides		LDL-C		Non-HDL-C		VLDL-C	
Result Category			n	Col%	n	Col%	n	Col%	n	Col%	n	Col%	n	Col%
Total			1636997		453770		424999		405542		201189		151497	
All Age Groups	Elevated	Abnormal	254 052	15.5	51 811	11.4	108 267	25.5	38 697	9.5	29 435	14.6	25842	17.1
		Borderline	308 737	18.9	107 557	23.7	102 858	24.2	56 173	13.9	42 149	20.9	.	
		Healthy	1 074 208	65.6	294 402	64.9	213 874	50.3	310 672	76.6	129 605	64.4	125655	82.9
9-11 Years	Elevated	Abnormal	45 925	15.9	8415	9.7	23 515	31.5	5254	7.4	3854	12.4	4887	19.7
		Borderline	55 312	19.2	21 484	24.8	18 111	24.3	8981	12.6	6736	21.6	.	
		Healthy	187 221	64.9	56 606	65.4	33 030	44.2	57 194	80.1	20 530	66.0	19861	80.3
12-16 Years	Elevated	Abnormal	83 720	14.7	14 981	9.2	39 092	26.6	11 198	8.0	9067	12.9	9382	18.2
		Borderline	102 773	18.0	34 983	21.6	36 353	24.8	17 520	12.5	13 917	19.9	.	
		Healthy	383 813	67.3	112 364	69.2	71 428	48.6	110 919	79.4	47 043	67.2	42059	81.8
17-21 Years	Elevated	Abnormal	124 407	16.0	28 415	13.9	45 660	22.4	22 245	11.4	16 514	16.5	11573	15.4
		Borderline	150 652	19.4	51 090	24.9	48 394	23.8	29 672	15.3	21 496	21.5	.	
		Healthy	503 174	64.7	125 432	61.2	109 416	53.8	142 559	73.3	62 032	62.0	63735	84.6

Lipid screening tests include total cholesterol, triglycerides, low-density lipoprotein cholesterol (LDL-C), non-high-density lipoprotein cholesterol (non-HDL-C), very low-density lipoprotein cholesterol (VLDL-C), as identified in IQVIA through key word search. Lipid measurements were considered elevated if ≥ 1 of the following was identified: TC ≥ 170 mg/dL, LDL-C ≥ 110 mg/dL, VLDL-C ≥ 31 mg/dL, non-HDL-C ≥ 120 mg/dL, or triglycerides ≥ 75 mg/dL (9 years) or ≥ 90 mg/dL (10-21 years). Among elevated values, levels were defined as abnormal if ≥ 1 of the following was identified: TC ≥ 200 mg/dL, LDL-C ≥ 130 mg/dL, VLDL-C ≥ 31 mg/dL, non-HDL-C ≥ 145 mg/dL, or triglycerides ≥ 100 mg/dL (9 years) or ≥ 130 mg/dL (10-21 years).

© 2024 Thompson-Paul AM et al. *JAMA Network Open.*

eTable 2. Comparison of Elevated Lipid Screening Results by Race and Ethnicity

					Lipid Screening Test Type											
			All		Total Cholesterol		Triglycerides		LDL-C		Non HDL-C		VLDL-C	
	Result Category		n	Col%	n	Col%	n	Col%	n	Col%	n	Col%	n	Col%
Total			163 6997		453 770		424 999		405 542		201 189		151 497	
**All Races and Ethnicities**	Elevated	Abnormal	254 052	15.5	51 811	11.4	108 267	25.5	38 697	9.5	29 435	15	25 842	17.1
		Borderline	308 737	18.9	107 557	23.7	102 858	24.2	56 173	13.9	42 149	21	.	
		Healthy	1 074 208	65.6	294 402	64.9	213 874	50.3	310 672	76.6	129 605	64	125 655	82.9
**Asian**	Elevated	Abnormal	10 500	16.2	2307	13.2	4052	24.6	1634	10.4	1662	16	845	16.2
		Borderline	13 619	21.0	4848	27.8	4175	25.3	2390	15.2	2206	22	.	
		Healthy	40 807	62.9	10 276	59.0	8261	50.1	11 692	74.4	6212	62	4366	83.8
**Black**	Elevated	Abnormal	19 964	10.7	5678	10.2	6418	13.5	4156	9.2	2292	12	1420	7.6
		Borderline	31 964	17.1	12 821	23.1	9055	19.1	6264	13.9	3824	20	.	
		Healthy	134 568	72.2	37 097	66.7	32 032	67.4	34 615	76.9	13 493	69	17 331	92.4
**Hispanic**	Elevated	Abnormal	1729	15.7	285	9.9	789	27.6	210	7.5	176	13	269	23.4
		Borderline	1945	17.6	625	21.7	701	24.5	347	12.4	272	20	.	
		Healthy	7359	66.7	1965	68.3	1366	47.8	2237	80.1	908	67	883	76.6
**Other**	Elevated	Abnormal	16 747	17.5	3051	11.8	7793	29.9	2428	10.0	1926	17	1549	20.5
		Borderline	18 426	19.3	6045	23.3	6460	24.8	3417	14.0	2504	22	.	
		Healthy	60 349	63.2	16 869	65.0	11 802	45.3	18 514	76.0	7156	62	6008	79.5
**Unknown**	Elevated	Abnormal	50 670	15.4	9480	10.8	22 098	25.6	7644	9.4	6783	15	4665	17.2
		Borderline	61 532	18.7	20 520	23.4	20 630	23.9	11 051	13.5	9331	20	.	
		Healthy	216 330	65.8	57 581	65.7	43 698	50.6	62 945	77.1	29 625	65	22 481	82.8
**White**	Elevated	Abnormal	154 442	16.2	31 010	11.7	67 117	27.3	22 625	9.6	16 596	15	17 094	18.6
		Borderline	181 251	19.1	62 698	23.7	61 837	25.2	32 704	13.9	24 012	21	.	
		Healthy	614 795	64.7	170 614	64.5	116 715	47.5	180 669	76.6	72 211	64	74 586	81.4

Lipid screening tests include low-density lipoprotein cholesterol (LDL-C), non-high-density lipoprotein cholesterol (non-HDL-C), very low-density lipoprotein cholesterol (VLDL-C), triglycerides, or total cholesterol (TC) as identified in IQVIA through key word search. Lipid measurements were considered elevated if ≥ 1 of the following was identified: TC ≥ 170 mg/dL, LDL-C ≥ 110 mg/dL, VLDL-C ≥ 31 mg/dL, non-HDL-C ≥ 120 mg/dL, or triglycerides ≥ 75 mg/dL (9 years) or ≥ 90 mg/dL (10-21 years). Among elevated values, levels were defined as abnormal if ≥ 1 of the following was identified: TC ≥ 200 mg/dL, LDL-C ≥ 130 mg/dL, VLDL-C ≥ 31 mg/dL, non-HDL-C ≥ 145 mg/dL, or triglycerides ≥ 100 mg/dL (9 years) or ≥ 130 mg/dL (10-21 years).

© 2024 Thompson-Paul AM et al. *JAMA Network Open.*
