# Supplementary material for: Pediatric Lipid Screening Prevalence Using Nationwide Electronic Medical Records
Source: JAMA Netw Open. 2024 Jul 23;7(7):e2421724. doi: 10.1001/jamanetworkopen.2024.21724 (PMC11267408; doi:10.1001/jamanetworkopen.2024.21724)
Supplement: Supplement 2. — Data Sharing Statement [file jamanetwopen-e2421724-s002.pdf]

Data Sharing Statement

Thompson-Paul. Pediatric Lipid Screening Prevalence Using Nationwide Electronic Medical Records. *JAMA Netw Open*. Published July 23, 2024.
doi:10.1001/jamanetworkopen.2024.21724

Data
Data available: No

Additional Information
Explanation for why data not available: IQVIA AEMR is a commercially available data asset. CDC is not allowed to share the data with external entities. Data must be requested and purchased directly from IQVIA.
